# Delta-9-tetrahydrocannabinol protects against MPP^+^ toxicity in SH-SY5Y cells by restoring proteins involved in mitochondrial biogenesis

**DOI:** 10.18632/oncotarget.10314

**Published:** 2016-06-27

**Authors:** Marie-Louise Zeissler, Jordan Eastwood, Kieran McCorry, C. Oliver Hanemann, John P. Zajicek, Camille B. Carroll

**Affiliations:** ^1^ Plymouth University Peninsula Schools of Medicine and Dentistry, Plymouth, PL6 8BU, United Kingdom; ^2^ School of Medicine, Medical and Biological Sciences, University of St Andrews, North Haugh, St Andrews, KY16 9TF, United Kingdom; ^3^ Plymouth University Peninsula Schools of Medicine and Dentistry, Plymouth, PL6 8BX, United Kingdom

**Keywords:** Δ^9^-tetrahydrocannabinol, peroxisome proliferator-activated receptor, MPP^+^, mitochondrial biogenesis, Parkinson's disease

## Abstract

Proliferator-activated receptor γ (PPARγ) activation can result in transcription of proteins involved in oxidative stress defence and mitochondrial biogenesis which could rescue mitochondrial dysfunction in Parkinson's disease (PD). The PPARγ agonist pioglitazone is protective in models of PD; however side effects have limited its clinical use. The cannabinoid Δ^9^-tetrahydrocannabinol (Δ^9^-THC) may have PPARγ dependent anti-oxidant properties. Here we investigate the effects of Δ^9^-THC and pioglitazone on mitochondrial biogenesis and oxidative stress. Differentiated SH-SY5Y neuroblastoma cells were exposed to the PD relevant mitochondrial complex 1 inhibitor 1-methyl-4-phenylpyridinium iodide (MPP^+^). We found that only Δ^9^-THC was able to restore mitochondrial content in MPP^+^ treated SH-SY5Y cells in a PPARγ dependent manner by increasing expression of the PPARγ co-activator 1α (PGC-1α), the mitochondrial transcription factor (TFAM) as well as mitochondrial DNA content. Co-application of Δ^9^-THC with pioglitazone further increased the neuroprotection against MPP^+^ toxicity as compared to pioglitazone treatment alone. Furthermore, using lentiviral knock down of the PPARγ receptor we showed that, unlike pioglitazone, Δ^9^-THC resulted in a PPARγ dependent reduction of MPP^+^ induced oxidative stress. We therefore suggest that, in contrast to pioglitazone, Δ^9^-THC mediates neuroprotection via PPARγ-dependent restoration of mitochondrial content which may be beneficial for PD treatment.

## INTRODUCTION

Parkinson's disease (PD) is a neurodegenerative disorder in which the progressive loss of dopaminergic neurons within the substantia nigra *pars compacta* leads to neurochemical imbalance in the basal ganglia resulting in motor dysfunction. Although PD can initially be managed through dopamine replacement therapy, there are currently no treatment strategies available to halt the progression of the disease. Activation of the peroxisome proliferator activated receptor gamma (PPARγ) by the specific thiazolidinedione (TZD) agonists rosiglitazone and pioglitazone has been found protective in both animal and cell culture models of PD [1–6]. Furthermore, the presence of neuronal PPARγ receptors has recently been reported in the substantia nigra *pars compacta* of non-human primates [7]. The PPARγ receptor is a ligand activated nuclear receptor that initiates transcription of genes containing a PPAR response element (PPRE) in their promoter region [8]. So far, the neuroprotective properties of PPARγ agonists in PD have been attributed to an induction of anti-inflammatory responses [3, 5, 9] and induction of genes involved in oxidative stress defence such as superoxide dismutase 1 (SOD1) and catalase [2]. However, activation of the PPARγ receptor can also regulate the *de novo* synthesis of mitochondria [10, 11] by inducing the expression of PPARγ coactivator-1α (PGC-1α) as well as the mitochondrial transcription factor A (TFAM), both of which are key regulators of mitochondrial biogenesis [11, 12]. This is particularly interesting as a meta-analysis of 9 genome wide expression studies demonstrated that gene sets controlled by PGC-1α were down-regulated in PD patients [13]. Restoration of PGC-1α levels has been shown to protect against complex 1 inhibition as well as in genetic models of PD such as A53T mutation of α-synuclein [13–15] and Park2 mutations [16]. Furthermore, the TZD rosiglitazone can restore mitochondrial content in differentiated SH-SY5Y cells treated with the complex 1 inhibitor rotenone as well as PTEN-induced putative kinase 1 (PINK1) knock down cells [6]. Collectively this evidence indicates that the ability to restore PGC-1α and thereby mitochondrial content may provide a novel treatment strategy in PD and there is some evidence that this may be achieved by targeting the PPARγ receptor.

However, TZDs such as rosiglitazone and pioglitazone are known to cause adverse effects in humans which have resulted in their withdrawal from clinical use in several European countries [17] thereby creating a need for non-TZD activators of the receptor to be investigated further. One such compound may be the phytocannabinoid, D^9^-tetrahydrocannabinol (D^9^-THC), which has been found protective in 6-hydroxydopamine (6-OHDA) lesioned rats [18, 19] and differentiated SH-SY5Y cells treated with the mitochondrial complex 1 inhibitor 1-methyl-4-phenylpyridinium iodide (MPP^+^) through a mechanism involving the PPARγ receptor, independent of cannabinoid receptors 1 and 2 [20].

Reduction in mitochondrial complex 1 activity is known to be a key feature in sporadic PD [21] and complex 1 inhibitors are therefore widely used as a model for mitochondrial dysfunction in PD [22]. We used MPP^+^ as a means to model PD-associated mitochondrial dysfunction in differentiated SH-SY5Y cells to further investigate the PPARγ mediated anti-oxidant effect of D^9^-THC. The suitability of differentiated human dopaminergic SH-SY5Y cells in Parkinson's disease research is still a subject of debate with some arguing for differentiation [23–25] and some against [26, 27]. However, differentiated cells are susceptible to MPP^+^ and express the required dopamine and noradrenalin transporters for uptake of the neurotoxin [24]. Furthermore, differentiation leads to a reduction in cell proliferation and the induction of a predominantly mature dopaminergic-like neurotransmitter phenotype [23, 24]. We therefore used differentiated SH-SY5Y cells in our study.

The primary aim of this study was to determine the downstream pathway through which D^9^-THC-mediated PPARγ activation leads to neuroprotection in our model and whether this pathway is similar to that of pioglitazone.

## RESULTS

### SOD1 and catalase expression are not affected by Δ^9^-THC and pioglitazone

**Figure 1:**
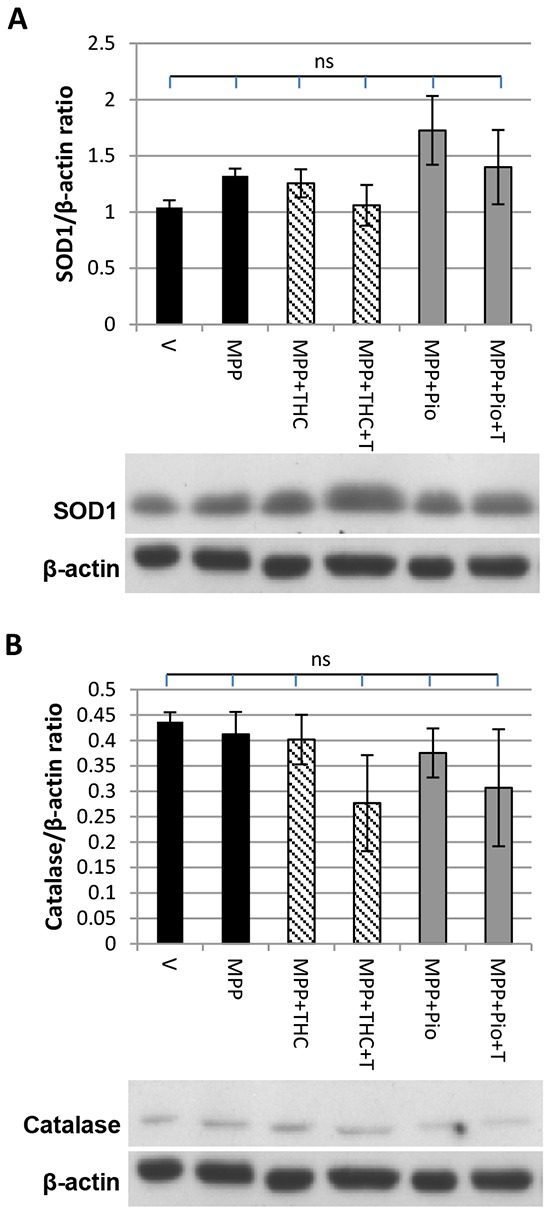
SOD1 and catalase expression Western blot showing the effect of MPP^+^, Δ^9^-THC and pioglitazone (pio) on **A.** SOD1 and **B.** catalase expression. Protein data were corrected to β-actin levels which served as loading control. Each bar represents the mean ± SEM from 3 independent experiments (one-way ANOVA with Tukey HSD post-hoc test, no significant difference).

Western blots were performed to investigate whether stimulation of PPARγ with Δ^9^-THC (10 μM) or pioglitazone (5 μM) led to an induction of the expression of its transcriptional targets SOD1 or catalase. SOD1 (Figure 1A) and catalase (Figure 1B) expression were unaffected by 7 mM MPP^+^ treatment and remained unchanged by co-application of 10 μM Δ^9^-THC, 5 μM pioglitazone or co-application of T0070907.

### Δ^9^-THC but not pioglitazone induces mitochondrial biogenesis

**Figure 2:**
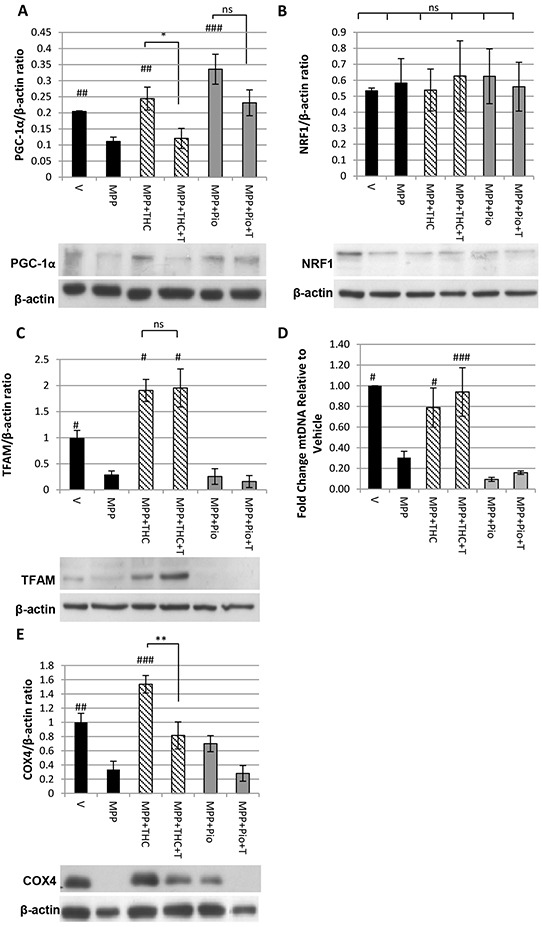
Δ^9^-THC but not pioglitazone induces mitochondrial biogenesis Western blot showing the effect of MPP^+^, Δ^9^-THC, pioglitazone (pio) and T0070907 (T) on **A.** PGC-1α expression (n = 5, one-way ANOVA with Scheffe post-hoc test), **B.** NRF1 expression (n = 5, one-way ANOVA with Tukey HSD post-hoc test) and **C.** TFAM expression (n = 3, one-way ANOVA with Tukey HSD post-hoc test). **D.** QPCR on genomic DNA extracts to determine the ratio between mitochondrial and genomic DNA. Cells were treated with MPP^+^, Δ^9^-THC, pioglitazone (pio) and T0070907 (T) (n = 3, one-way ANOVA with Tukey HSD post-hoc test). **E.** Western blot showing the effect of MPP^+^, Δ^9^-THC, pioglitazone (pio) and T0070907 (T) on COX4 expression (n = 4, one-way ANOVA with Tukey HSD post-hoc test). Protein data were corrected to β-actin levels which served as loading control. Each bar represents the mean ± SEM (#p<0.05, ##p<0.01, ###p<0.0001 vs. MPP^+^).

Western blots were carried out to investigate whether 10 μM Δ^9^-THC and 5 μM pioglitazone led to increased expression of the master regulator of mitochondrial biogenesis PGC-1α. Figure 2A shows that protein levels of PGC-1α were down-regulated compared to untreated vehicle control upon 7 mM MPP^+^ treatment and restored by 10 μM Δ^9^-THC. This restoration was completely reversed by the addition of 10 μM T0070907, indicating that it was a PPARγ-mediated effect. Pioglitazone (5 μM) co-application also led to a significant increase in the PGC-1α expression compared to MPP^+^ alone (Figure 2A). Addition of T0070907 (5 μM) did not significantly block up-regulation of PGC-1α expression.

The transcription factor nuclear respiratory factor 1 (NRF1) regulates the expression of nuclear encoded mitochondrial genes and is transcriptionally regulated by PGC-1α [28]. No changes were detected in NRF1 expression with 7 mM MPP^+^, 10 μM Δ^9^-THC or 5 μM pioglitazone treatment (Figure 2B).

Sufficient levels of the mitochondrial transcription factor TFAM, as well as mtDNA replication are crucial for the *de novo* synthesis of mitochondria. TFAM expression was significantly reduced by 7 mM MPP^+^ treatment compared to vehicle treated cells and was fully restored by co-application of 10 μM Δ^9^-THC; this restorative effect was not reversed by addition of 10 μM T0070907. Pioglitazone (5 μM) did not restore TFAM levels (Figure 2C). QPCR was carried out to determine the mtDNA content relative to nuclear DNA content. Similar to TFAM expression levels, mtDNA content was reduced by 7 mM MPP^+^ treatment compared to untreated vehicle control and only restored by co-application of 10 μM Δ^9^-THC in a PPARγ independent manner (Figure 2D).

Consequently, to determine whether Δ^9^-THC had an effect on mitochondrial biogenesis, the expression of COX4, a subunit of the electron transport chain, was measured by western blotting. Figure 2E shows that 7 mM MPP^+^ treatment significantly reduced COX4 levels compared to untreated vehicle control. Co-application of 10 μM Δ^9^-THC with MPP^+^ completely restored COX4 levels, which was reversed by the PPARγ antagonist T0070907 (10 μM) indicating that the cannabinoid induces mitochondrial biogenesis in a PPARγ-dependent manner. 5 μM Pioglitazone had no significant effect on MPP^+^ mediated down-regulation of COX4 expression.

### Δ^9^-THC and pioglitazone act through divergent pathways

**Figure 3:**
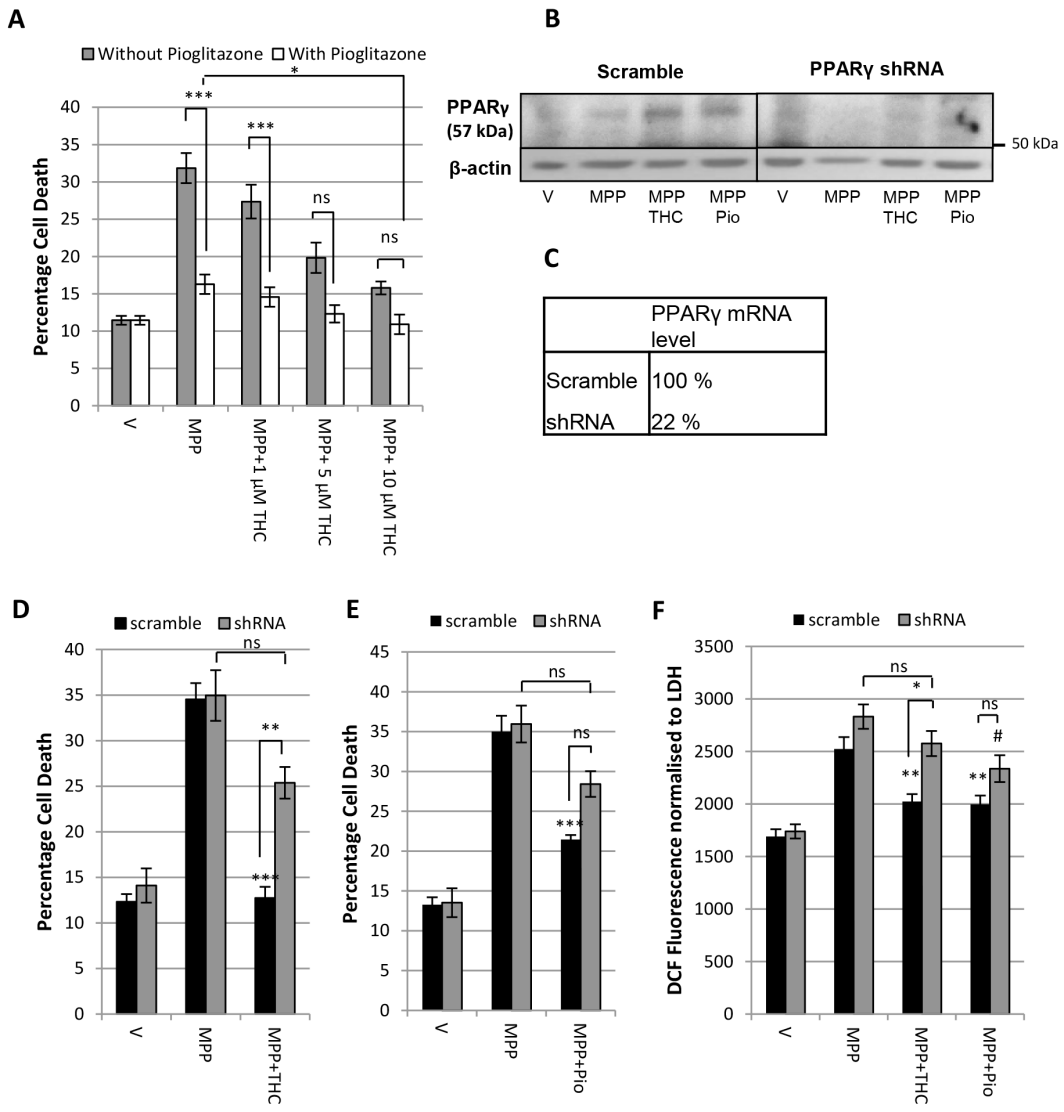
Δ^9^-THC and pioglitazone act through divergent pathways **A.** LDH assay demonstrating the additive protective effect of 5 μM pioglitazone (pio) with increasing concentrations Δ^9^-THC against 7 mM MPP^+^ toxicity. **B.** Western Blot and **C.** QPCR showing the PPARγ protein and mRNA levels of SH-SY5Y cells infected with lentivirus containing a non-specific scramble sequence and PPARγ shRNA respectively. **D.** LDH assay showing that the PPARγ shRNA construct prevents protection afforded by 10 μM Δ^9^-THC against 7mM MPP^+^ but not **E.** 5 μM pioglitazone. **F.** DCFDA oxidative stress assay demonstrating that whereas knock down of PPARγ prevents 10 μM Δ^9^-THC mediated reduction in oxidative stress, PPARγ knock down does not prevent 5 μM pioglitazone-mediated reduction in oxidative stress. Each bar represents the mean ± SEM from 3 independent experiments (one-way ANOVA with Tukey HSD post-hoc test,*p < 0.05, **p < 0.001, ***p < 0.0001 vs. scramble MPP^+^; #p < 0.05 vs. shRNA MPP^+^).

Given the differences in downstream effects of Δ^9^-THC and pioglitazone it is possible that the protective effects of the two compounds may be additive when applied in combination. To test this hypothesis, the protective effects of increasing concentrations of Δ^9^-THC against the toxicity of 7 mM MPP^+^ were assessed on its own and in combination with 5 μM pioglitazone (Figure 3A). The concentration of pioglitazone was chosen as it resulted in the highest protective effect in this model [20]. Increasing concentrations of Δ^9^-THC on its own led to dose dependent protection against MPP^+^. Pioglitazone (5 μM) alone also significantly reduced MPP^+^ induced cell death, a protective effect that was increased further by co-application of 10 μM Δ^9^-THC.

This finding suggests that either the compounds have complementary activity on the same protective pathway, or they act synergistically via divergent downstream pathways. To clarify this further, a PPARγ knock down was utilised using a lenti-virus to infect SH-SY5Y cells with PPARγ shRNA. The level of knock down was validated by western blotting and QPCR (Figure 3B, 3C). PPARγ mRNA levels were reduced to 22 % relative to the scramble sequence and PPARγ protein levels were reduced in knock down samples.

Figure 3D shows that SH-SY5Y cells infected with scramble sequence were protected by 10 μM Δ^9^-THC against 7 mM MPP^+^ leading to a significant reduction in cell death. This confirmed that lentiviral infection itself did not influence the toxicity of MPP^+^ or the protective potential of Δ^9^-THC. There was no significant difference in percentage cell death between PPARγ shRNA treated cells with MPP^+^ alone and in combination with Δ^9^-THC showing that PPARγ knock down can prevent Δ^9^-THC-mediated neuroprotection. This was further confirmed by a significant difference between scramble shRNA and PPARγ shRNA infected cells treated with Δ^9^-THC and MPP^+^. Similarly, cells infected with scramble shRNA could be rescued from cell death caused by 7 mM MPP^+^ with co-application of 5 μM pioglitazone (Figure 3E). However, cells infected with PPARγ shRNA were neither significantly protected from MPP^+^ toxicity when co-treated with pioglitazone nor was there a significant difference between scramble and PPARγ shRNA infected cells treated with pioglitazone.

We previously demonstrated that Δ^9^-THC reduces oxidative stress in a PPARγ dependent manner [20]. We therefore investigated whether PPARγ knock down could prevent Δ^9^-THC and pioglitazone mediated anti-oxidant effects as assessed by DCF fluorescence. In cells infected with scramble sequence, 10 μM Δ^9^-THC treatment led to a significant reduction in oxidative stress compared to MPP^+^ (7 mM) treatment alone. In the shRNA knock down cells, oxidative stress was increased with MPP^+^ but there was no significant reduction with Δ^9^-THC treatment confirming that Δ^9^-THC-mediated reduction in oxidative stress is indeed mediated by PPARγ receptor activation. Similarly, Figure 3F shows that 5 μM pioglitazone significantly reduced reactive oxygen species (ROS) production in cells infected with scramble sequence. Lentiviral knock down of PPARγ was not able to block the reduction in ROS production caused by pioglitazone treatment completely.

**Figure 4:**
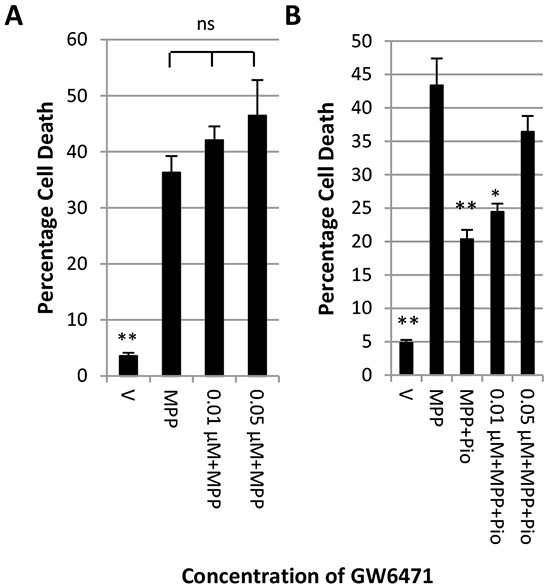
Inhibition of PPARα blocks the protective effect of pioglitazone LDH assay showing the effect of increasing concentrations of PPARα antagonist GW6471 on **A.** the toxicity of 7 mM MPP^+^ and **B.** the protective effect of pioglitazone. Each bar represents the mean ± SEM of quadruplicate measurements from 3 independent experiments (one-way ANOVA with Tukey HSD post-hoc test; *p < 0.05, **p < 0.001 vs. MPP^+^).

The evidence presented here indicates that pioglitazone may not act exclusively through the PPARγ receptor. Pioglitazone has been shown to activate the human PPARα receptor [29]. We therefore tested whether the specific PPARα receptor antagonist GW6471 could reverse the protective effect of pioglitazone. The LDH assay in Figure 4A shows that GW6471 at concentrations of up to 0.05 μM did not increase MPP^+^ toxicity. Furthermore, 0.05 μM GW6471 reversed pioglitazone mediated neuroprotection against MPP^+^ (Figure 4B).

## DISCUSSION

Δ^9^-THC has been found neuroprotective in PD and this neuroprotective effect has in the past mainly been attributed to the cannabinoid's innate anti-oxidant structure, independent of cannabinoid receptor activity [18, 30, 31]. We further confirmed that the neuroprotective action of Δ^9^-THC is not cannabinoid receptor 1 (CB1 receptor) mediated and identified the PPARγ receptor as a potential target through which Δ^9^-THC may exert its anti-oxidant effects [20] thereby making it unlikely that Δ^9^-THC has an effect on mitochondrial CB1 receptors [32] in our model, the presence of which has not yet been demonstrated in SH-SY5Y cells. The primary aim of the present study was to further assess how PPARγ receptor activation by Δ^9^-THC leads to neuroprotection and whether this mechanism is similar to that of the PPARγ agonist pioglitazone. Our data indicate that rather than reducing oxidative stress by PPARγ-regulated expression of the antioxidant enzymes SOD1 and catalase, Δ^9^-THC induces PPARγ-mediated mitochondrial biogenesis whilst pioglitazone, whose protective effect is most likely only partially driven by PPARγ, does not. Indeed our data suggest that Δ^9^-THC can add to the neuroprotective effect of pioglitazone.

In PD, mitochondrial function is compromised leading to increased production of reactive oxygen species thereby causing irreversible damage to proteins [33], lipids [34] and DNA [35] resulting in cell death [22, 36, 37]. Although PPARγ stimulation can play a role in modulating cellular redox balance by controlling the expression of NRF2 [38] as well as the anti-oxidant enzymes superoxide dismutase 1 (SOD1) and catalase [39, 40], it is not clear whether PPARγ-mediated protection against oxidative stress in the MPP^+^ model of PD involves SOD1 and catalase as one group demonstrated increased activity and expression of the enzymes [2] in response to rosiglitazone treatment whilst another found that SOD1 activity and expression remained unchanged [41]. We found SOD1 and catalase expression to be unchanged by MPP^+^, Δ^9^-THC or pioglitazone treatment, suggesting that these enzymes were not involved in Δ^9^-THC or pioglitazone mediated neuroprotection. The PPARγ receptor is able to regulate the expression of a vast variety of target genes. It is now increasingly recognised that the extent of expression of these genes can vary depending on the agonist used as they may alter intracellular factors such as the availability of specific co-activators [42], interaction with other nuclear receptors [43] as well as receptor phosphorylation, ubiquitination and sumoylation [44, 45]. This might explain why, despite being PPARγ target genes, neither Δ^9^-THC nor pioglitazone had any effect on catalase or SOD1 expression. Complex 1 inhibition, which has been associated with sporadic PD [21, 46, 47], leads to reduced expression of proteins necessary for the *de novo* synthesis of mitochondria. Specifically, PGC-1α and TFAM expression has been shown to be decreased in MPP^+^ treated SH-SY5Y cells resulting in reduced mitochondrial content, closely mirroring our findings [48, 49]. Reduced mitochondrial content seems to be a common phenomenon in patients with sporadic PD [13] as well as familial models of PD including neurons overexpressing the A53T mutation of α-synuclein [15] and neurons expressing mutant leucine rich repeat kinase 2 (LRRK2) [50]. Furthermore, transcriptional activity of PGC-1α is regulated by parkin and therefore mutations that impair the functionality of parkin also impact mitochondrial biogenesis [16, 51, 52]. Hence, treatments that induce mitochondrial biogenesis may be of particular benefit in PD.

PGC-1α is a target of PPARγ activation with the potential ability to reduce oxidative stress [12]. As well as co-activating the PPARγ receptor and thereby enhancing the transcriptional activity of PPARγ, PGC-1α is known as the master-regulator of mitochondrial biogenesis as it orchestrates the expression of transcription factors TFAM and NRF1 which are responsible for transcription of mitochondrial as well as nuclear encoded mitochondrial genes respectively [15, 28]. We therefore hypothesised that, by activating PPARγ, pioglitazone and Δ^9^-THC might induce the expression of PGC-1α which may in turn stimulate the transcription of components essential for mitochondrial biogenesis. Restoration of PGC-1α expression can reverse reduction in mitochondrial content which has been found protective in models using complex 1 inhibitors such as 1-methyl-4-phenyl-1,2,3,6-tetrahydropyridine (MPTP) and rotenone [13, 14], A53T mutations of α-synuclein [13, 15] and α-synuclein oligomerisation [53].

Both Δ^9^-THC and pioglitazone treatment led to restoration of PGC-1α levels in our MPP^+^ treated cells. However, only Δ^9^-THC was able to simultaneously restore TFAM and mtDNA levels leading to increased COX4 expression which we used as a marker for mitochondrial biogenesis. There is evidence that restoration of TFAM can reverse mitochondrial deficits and increase mitochondrial density in MPP^+^ treated SH-SY5Y cells [48, 49]. TFAM may play a direct role in the stabilisation and maintenance of mtDNA as TFAM knock down leads to accumulation of mtDNA mutations [54] whilst its overexpression increases mtDNA content [55] as well as expression of mitochondria encoded electron transport chain subunits [55, 56]. We suggest that whilst reduced TFAM expression can become rate limiting to mitochondrial biogenesis, it requires the presence of PGC-1α to co-ordinate the production of new mitochondria as it co-activates NRF1 mediated transcription of nuclear encoded mitochondrial subunits [28]. Therefore, pioglitazone was unable to induce mitochondrial biogenesis as, despite increasing PGC-1α expression, it lacked the ability to restore TFAM levels. It is not clear whether increasing mitochondrial density and mtDNA levels would be protective long term as newly produced mitochondria might still be vulnerable to oxidative damage. Therefore, future experiments may include investigating rates of DNA oxidation in response to Δ^9^-THC treatment.

Whilst we have identified a potential mechanism through which Δ^9^-THC can be anti-oxidant, namely by increasing mitochondrial biogenesis, the mechanism which mediates the anti-oxidant effect of pioglitazone remains unclear. Lipophilicity is thought to be a key determinant of the biological activity of drugs as it plays a role in their bioavailability. Both pioglitazone and Δ^9^-THC are lipophilic compounds with a log P value of 4 and 6.97 respectively [57, 58]. Whilst in theory higher lipophilicity may increase the ability of a compound to cross membranes and therefore reach an internal target receptor, this is also dependent on other factors such as charge and size of the compound. It is, however, unlikely that Δ^9^-THC is more potent at activating PPARγ solely because of its higher lipophilicity as some studies looking at the relationship between lipophilicity and PPARγ activation within the TZD family found no correlation between lipophilicity and PPARγ activity [59].

Interestingly, pioglitazone has been shown to bind and activate PPARα in the micromolar range [29] which can also induce PGC-1α expression and reduce oxidative stress [60]. This might explain the PPARγ independent increase in PGC-1α expression in response to pioglitazone treatment. Our preliminary results indicate that pioglitazone could, at least in part, act through activation of the PPARα receptor as the protective effect of pioglitazone can be reversed with the specific inhibitor GW6471. Furthermore, PPARα activation by fenofibrate and palmitoylethanolamide (PEA) has been found neuroprotective against 6-OHDA and MPTP mediated toxicity respectively [61–63]. Alternatively, like other thiazolidinediones, pioglitazone could have other receptor independent anti-oxidant effects [38, 41, 64].

Our results show that whilst both D^9^-THC and pioglitazone reduced oxidative stress and restored PGC-1α levels, only D^9^-THC had the ability to induce TFAM expression and restore mitochondrial DNA (mtDNA) levels leading to increased cytochrome c oxidase subunit 4 (COX4) expression which had been almost completely diminished by MPP^+^. Furthermore, lentiviral knock down experiments indicated that only the D^9^-THC mediated protective effect was exclusively mediated by PPARγ. Co-application experiments with pioglitazone show that Δ^9^-THC adds to the neuroprotective effect of pioglitazone thereby suggesting that the two compounds act through divergent pathways and provide the first evidence that the cannabinoid could be an alternative protective strategy to TZDs by restoring mitochondrial content in a PPARγ-dependent manner. Although not assessed in the current study, it is possible that Δ^9^-THC would also have neuroprotective effects additional to those postulated for monoamine oxidase inhibitors (MAOIs), widely used in clinical practice, whose mode of action is thought to be anti-apoptotic, largely independent of their MAO effects [65]. This raises the possibility of these drugs being utilised in combination to maximise any clinical neuroprotective benefit and merits further investigation.

In recent years, PPARγ agonists have been considered as promising therapeutic agents for the treatment of PD [66]. However, the clinical potential of classical TZD-based PPAR agonists is limited with rosiglitazone having been withdrawn from clinical use in Europe due to its cardiovascular toxicity and pioglitazone being banned in Germany and France due to an associated risk for the development of bladder cancer [17]. By contrast, Δ^9^-THC is generally well tolerated by PD patients [67] and may therefore represent an alternative worthy of consideration. Furthermore, the ability of Δ^9^-THC to induce mitochondrial biogenesis is interesting as decreased mitochondrial content has been associated with familial as well as sporadic cases of PD.

## MATERIALS AND METHODS

Chemicals were purchased from Sigma-Aldrich Chemicals (Dorset, UK) unless stated otherwise.

### Culture of neuroblastoma cells

Human neuroblastoma cells (SH-SY5Y) were obtained from the European collection of cell cultures (ECACC) and grown in Dulbecco's modified Eagle's medium (Invitrogen, Paisley, UK) containing 10% (v/v) foetal bovine serum (FBS) (PAA, Yeovil, UK), glutamine, 4.5 g/l glucose, 1 ml uridine (25 mg/ml), 5 ml pyruvate, 25 units/ml penicillin and 25 μg/ml streptomycin, and incubated at 37°C in a humidified atmosphere of 5% CO_2_ and 95% air. For experiments, cells were seeded in 6-well dishes (200,000 cells/well) or 96-well plates (10,000 cells/well). After allowing the cells to attach for 24 hours, they were treated with 10 μM retinoic acid for 5 days to promote differentiation to a neuronal phenotype after which the cells were 90% confluent. Medium was changed every 48 hrs.

### Cell treatments

All treatments were made up in retinoic acid supplemented medium (10 μM). After differentiation, cells were treated with 7 mM MPP^+^ as well as 10 μM D^9^-THC or 5 μM pioglitazone for 48 hours. These concentrations were previously found to be protective in our cell culture model [20]. The minimum dose of the PPARγ inhibitor T0070907 required to completely reverse the protective effect of D^9^-THC and pioglitazone was used, 10 μM and 5 μM respectively (Carroll et al., 2012).

### Lentiviral knock down of PPARγ

Cells were seeded into 6 well plates and grown to 90% confluency. The cationic polymer polybrene was added to growth medium at a final concentration of 8 μg/ml. Pre-packaged high titre lentivirus containing PPARG shRNA 239215 (Open Biosystems, Fisher, Loughborough, UK) was defrosted at 37°C and 1 μl was added per well (multiplicity of infection = 31.8). Cells were incubated with the virus for 24 hours after which medium was replaced with selection medium containing 4 μg/ml puromycin. After 48 hours the concentration of puromycin was lowered to 2 μg/ml to ensure normal growth of cells which were kept at this concentration throughout all experiments. A lentivirus containing a non-specific scramble sequence was used as a control.

### LDH-release assay

To assess the cytotoxicity of the toxins under our experimental conditions, lactate dehydrogenase (LDH) release of cells grown and treated in 96-well plates was measured. Cell culture medium (50 μl) was used to analyse the LDH activity by measuring the oxidation of NADH at 450 nm as described in the manufacturer's protocol (Promega, Southampton, UK). The remaining cells were lysed at −80°C and LDH activity similarly measured to allow the percentage of cell death relative to the total number of cells to be calculated.

### Measurement of reactive oxygen species

SH-SY5Y cells were seeded into 96 well plates and treated for 48 hours as described above. The medium was removed and cells were incubated in serum free medium containing 10 μM 2′,7′-dichlorofluorescein diacetate (DCFDA) for 30 minutes. Cells were then washed 3 times with PBS after which the fluorescence was measured at Ex: 485 nm and Em: 535 nm. Surviving cells were lysed at −80°C and an LDH assay carried out. DCF fluorescence readings were then normalised to LDH absorbance to control for variations in cell number. Experiments were carried out 3 times in quadruplicate.

### Protein extraction and western blot analysis

Surviving cells were lysed for protein extraction 48 hours following exposure to treatments. Cells were washed with ice-cold PBS and protein extracted with NET-Triton buffer (150 mM NaCl, 5 mM EDTA (ethylenediaminetetraacetic acid), 10 mM Tris (tris(hydroxymethyl)aminomethane), pH 7.4, 1% Triton X-100) supplemented with complete protease inhibitor cocktail (Roche Diagnostics, Lewes, UK) according to the manufacturer's instructions.

The proteins were resolved by SDS/PAGE (10% gels) and blotted onto polyvinylidene fluoride (PVDF) membranes. Membranes were washed with tris-buffered saline (140 mM NaCl, 50 mM tris/HCl, pH 7.2) containing 0.1% Tween 20, 5% skimmed milk and 2% bovine serum albumin to block non-specific protein binding.

Membranes were incubated with primary antibody against PPARγ receptor (1:1000, 2435S New England Biolabs, Hitchin, UK) (57 kDa), SOD1 (1:1000, ab16831, Abcam, Cambridge, UK) (17 kDa), catalase (1:1000, ab16731, Abcam, Cambridge, UK) (60 kDa), PGC-1α (1:500, ab77210, Abcam, Cambridge, UK) (91 kDa), TFAM (1:1000, 8076, New England Biolabs, Hitchin, UK) (24 kDa), NRF1 (1:1000, ab55744, Abcam, Cambridge, UK) (54 kDa), COX4 (1:1000, ab33985, Abcam, Cambridge, UK) (15 kDa), in tris-buffered saline (140 mM NaCl, 50 mM tris/HCl, pH 7.2) containing 0.1% Tween 20 (TBST), 5% skimmed milk and 2% bovine serum albumin overnight at 4°C. PGC-1α antibody (1:500, ab77210, Abcam, Cambridge, UK) was incubated in TBST containing 5% bovine serum albumin overnight at 4°C. Subsequently membranes were washed 3 times in TBST and incubated with horseradish-peroxidase-conjugated secondary antibody (Bio-Rad, Hemel Hempstead, UK) for 1 h at room temperature. The protein bands were detected using ECL2 (Thermo Scientific, Rockford, US). Membranes were probed with β-actin (1:10000, ab8227, Abcam, Cambridge, UK) to control for loading. Samples were analysed from at least three separate experiments.

### Measuring mitochondrial DNA content

Cells were grown and treated in 6 well plates. Total DNA was extracted using the QIAamp DNA Mini Kit (QIAGEN, Crawley, UK) following the manufacturer's instructions for cultured cells. Changes in mtDNA content were then determined by QPCR. Specific primer pairs were designed to recognise a sequence from the mitochondrial genome (FW: CCCTATGTCGCAGTATCTGTCTTT; Rev: AGATGTGTTTAAGTGCTGTGGC) and the nuclear gene Pou5f1 (FW: CCTTCGCAAGCCCTCATTTC, Rev: TAGCCAGGTCCGAGGATCAA). QPCR was performed in a 50 μl reaction containing 25 μl 2x SYBR® GreenER™ mastermix, 1 μl/primer and 2 μl DNA. In a Biorad ICycler samples were heated to 50°C for 2 minutes followed by 95°C for 10 minutes. Samples were then cycled through 40 cylces of 95°C for 15 seconds and 63°C for 1 minute. A fluorescent signal was measured at Em: 520 nm when excited at Ex: 497 nm.

Each sample was run in triplicate. The ΔΔCT method was used to calculate fold changes in mtDNA content.

### Data analysis and statistical procedures

All numerical data are presented as mean ± 1 standard error of the mean. Each experiment was carried out at least 3 times. Statistical significance was determined with SPSS using one way analysis of variance (ANOVA) with a significance threshold of 95%, unless stated otherwise. To compare levels of significance between groups, an appropriate post-hoc test was chosen according to the assumptions of the test.
